# Long-lasting severe knee pain in a SLE patient after renal transplantation: what is the reason? A case report and literature review

**DOI:** 10.1007/s00296-021-05018-8

**Published:** 2021-10-19

**Authors:** Anna Masiak, Iga Kościńska, Beata Rutkowska, Zbigniew Zdrojewski

**Affiliations:** 1 Department of Internal Medicine, Connective Tissue Diseases and Geriatrics, Medical University of Gdansk, ul. Dębinki 7, 80-952 Gdańsk, Poland; 2 Department of Radiology, Medical University of Gdansk, Gdansk, Poland

**Keywords:** Arthralgia, Lupus nephritis, Kidney transplantation, Bone

## Abstract

Musculo-skeletal complaints in a patient suffering from systemic lupus, with co-existing chronic renal failure, undergoing immunosuppressive treatment after kidney transplantation, can have a varied etiology. The aim of this work was to present a case based review of differential diagnosis of knee pain in such a patient. A literature search was carried out using MEDLINE/PubMed, Google Scholar and EBSCO, with no time limit. We undertook a systematic review of the literature published in English, limited to full-text publications of original articles, letters to the editor, and case reports in peer-reviewed journals, for a discussion and analysis of studies reporting arthralgia in patients with lupus after kidney transplantation. We present a case report of a 45-year-old woman with lupus nephritis, after kidney transplantation, who started to complain of increasing pain in the knees, most pronounced at night and after physical activity approximately 2 years after transplantation. Extensive causal diagnostics were carried out, which revealed bilateral extensive regions of bone infarction in the femur and tibia, chondropathy, degenerative changes of medial meniscuses in the body and posterior horn as well as chondromalacia of the patella. Establishing the right diagnosis is crucial for implementing appropriate treatment.

## Introduction

Musculo-skeletal complaints in a systemic lupus erythematosus (SLE) patient after kidney transplantation can have a varied etiology and pose a diagnostic dilemma. Musculoskeletal complaints have been noted in up to 30% of patients receiving kidney transplants. In some patients, symptoms already occur before organ transplantation, and after this procedure, some of these problems persist, others improve, and new joint disorders may also develop [1].

Osteonecrosis is a common bone complication of SLE, which is reported in 4–15% lupus patients. The most common site of osteonecrosis is femoral head. Isolated symptomatic knee osteonecrosis in patients with SLE is a relatively rare complication which mimics the symptoms of lupus-associated non-erosive arthritis. In the differential diagnosis of knee pain, many other causes should also be considered, such as calcium–phosphate imbalance, osteomalacia, osteoporosis, osteoarthritis, or gout. Calcineurin inhibitor pain syndrome (CIPS) also known as immunosuppression-related bone marrow edema syndrome or post-transplant bone marrow edema syndrome is another potential cause of severe bone pain in patient treated with calcineurin inhibitor. The possible causes are numerous and usually multiple factors are present in each patient. Establishing the right diagnosis is crucial for implementing appropriate treatment. Elements of anamnesis such as character of pain (acute or chronic), aggravating and alleviating circumstances (whether pain is worse when first moving a joint or after prolonged use), time from the onset, time frame (new-onset or recurrent), morning predilection, together with laboratory findings and imaging examinations must be analyzed simultaneously to create a coherent picture of specific disorder.

Based on the case report of a 45-year old SLE patient with lupus nephritis after renal transplantation presenting chronic knee pain, we review the literature on the potential causes and differential diagnosis of knee pain and prevalence of knee avascular necrosis in this group of patients.

## Methods

### Search strategy

**Fig. 1:**
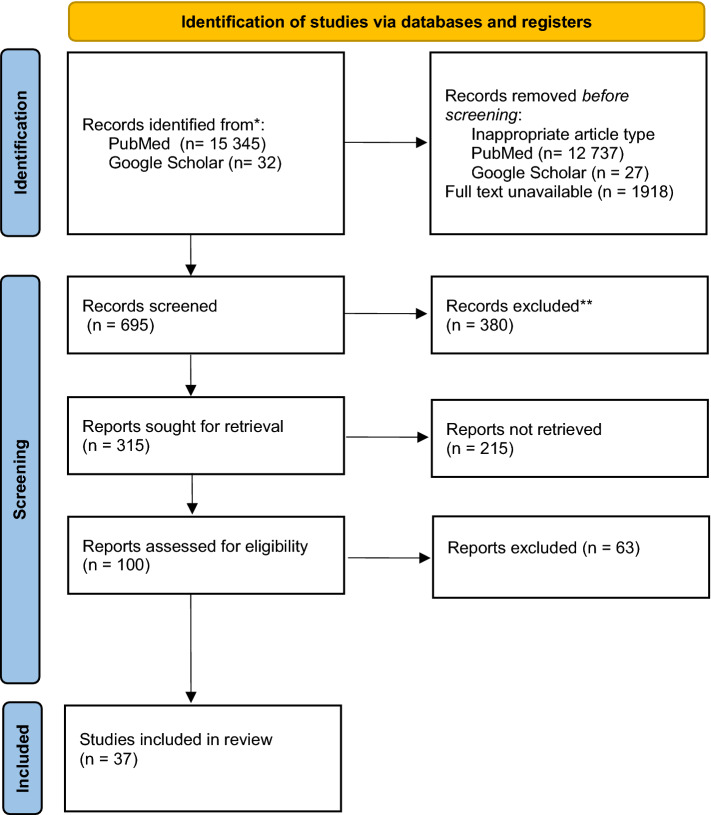
Search flow chart

A literature search for patients with lupus nephritis after renal transplantation suffering from chronic knee pain as well as the incidence of avascular necrosis of the knee joint in lupus patients was carried out using MEDLINE/PubMed, Google Scholar and EBSCO, with no time limit. The search was conducted using the following key words: “arthralgia knee” OR “knee pain” AND/OR “lupus” AND/OR “kidney transplantation” AND/OR “osteonecrosis”. Using a combination of these search terms, we undertook a systematic review of the literature published in English, limited to full-text publications of original articles, letters to the editor, and case reports in peer-reviewed journals, for a discussion and analysis of studies reporting causes of knee pain in patients with lupus after kidney transplantation (search strategy is presented in Fig. 1). The results are presented in Tables 1 and 2.

**Table 1:** Causes of musculoskeletal complaints (MSC) in patients after kidney transplantation

Author	Kart-Köseoglu [1]	Atallah [3]	Donmez [10]
Nr of patients in the study group	82	117	81 (57 with hip pain and 24 with knee pain)
Nr of pts complaining from MSC	Nd	95	81
Bone loss	Nd	78	Nd
Joint pain	24	63	Hips: 18 Knee: 9 degenerative joint disease; 7 chondromalacia, 6 meniscal tear, 6 ligament rupture
Skeletal muscle affection	Nd	21	Hips: 10 Knee: 3
Soft tissue affection	Nd	25	Hips: 13
Leg bone pain syndrome	Nd	7	Nd
Avascular osteonecrosis (AVN)	5	Nd	Hips: 26 Knee: 5 bone marrow oedema; 2 bone infarct
Gouty arthritis	2	Nd	0
Septic arthritis	1	Nd	0

*Nd *no data

**Table 2:** The prevalence of knee avascular necrosis (AVN) in patients with SLE

Author	Number of SLE patients	Number of cases with AVN	Number of cases (or joints) with knee AVN
Shigemura [16]	173	255 joints	141 joints
Ohtsuru [32]	300	5	1
Zhao [33]	3941	20	40
Sayarlioglu [15]	868	49	13
Ersin [34]	912	97	37
Gontero [12]	158	15	4
Gladman [35]	1729	234	86
Chinnadurai [36]	415	21	0
Dogan [37]	127	11	3
Nakamura [38]	126	207 joints	112 joints
Oinuma [39]	72	32	9
Kunyakham [40]	736	65	0

## Results

### Case presentation

**Fig. 2:**
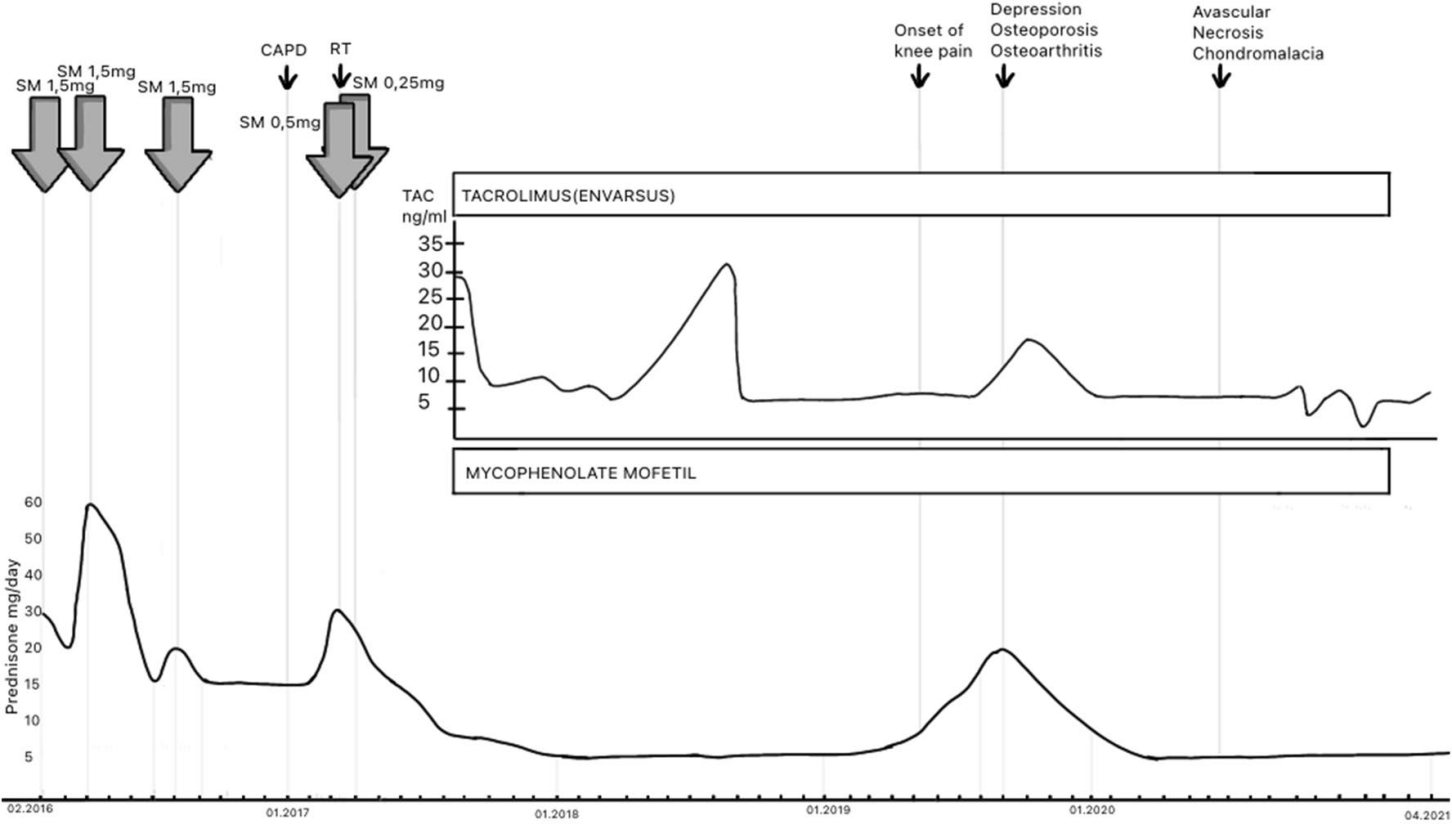
Clinical course and treatment of the patient with doses of steroids and tacrolimus though levels. *SM *methylprednisolone, *CAPD *continuous ambulatory peritoneal dialysis, *RT* renal transplantation;

**Fig. 3:**
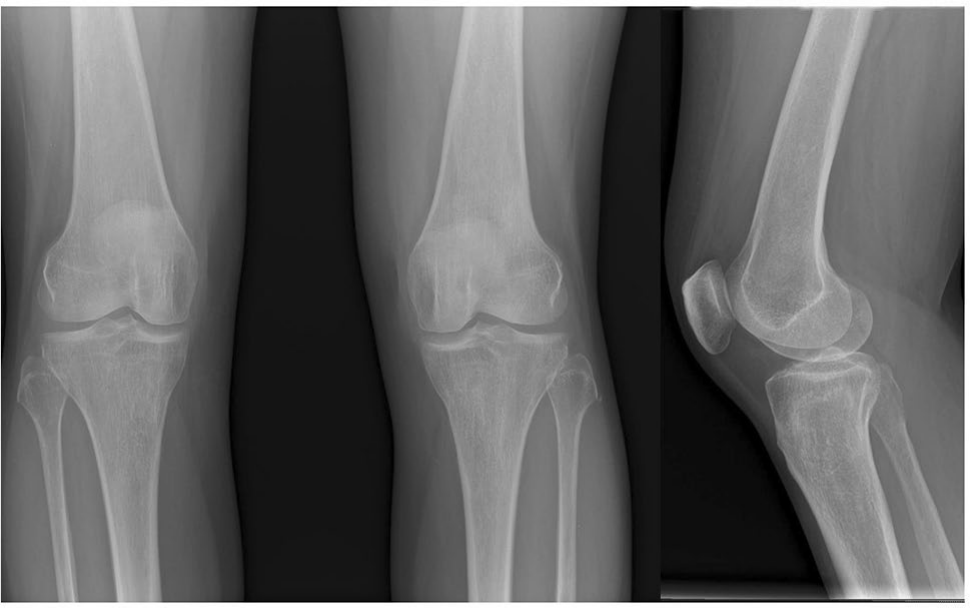
X-ray of the knees

**Fig. 4:**
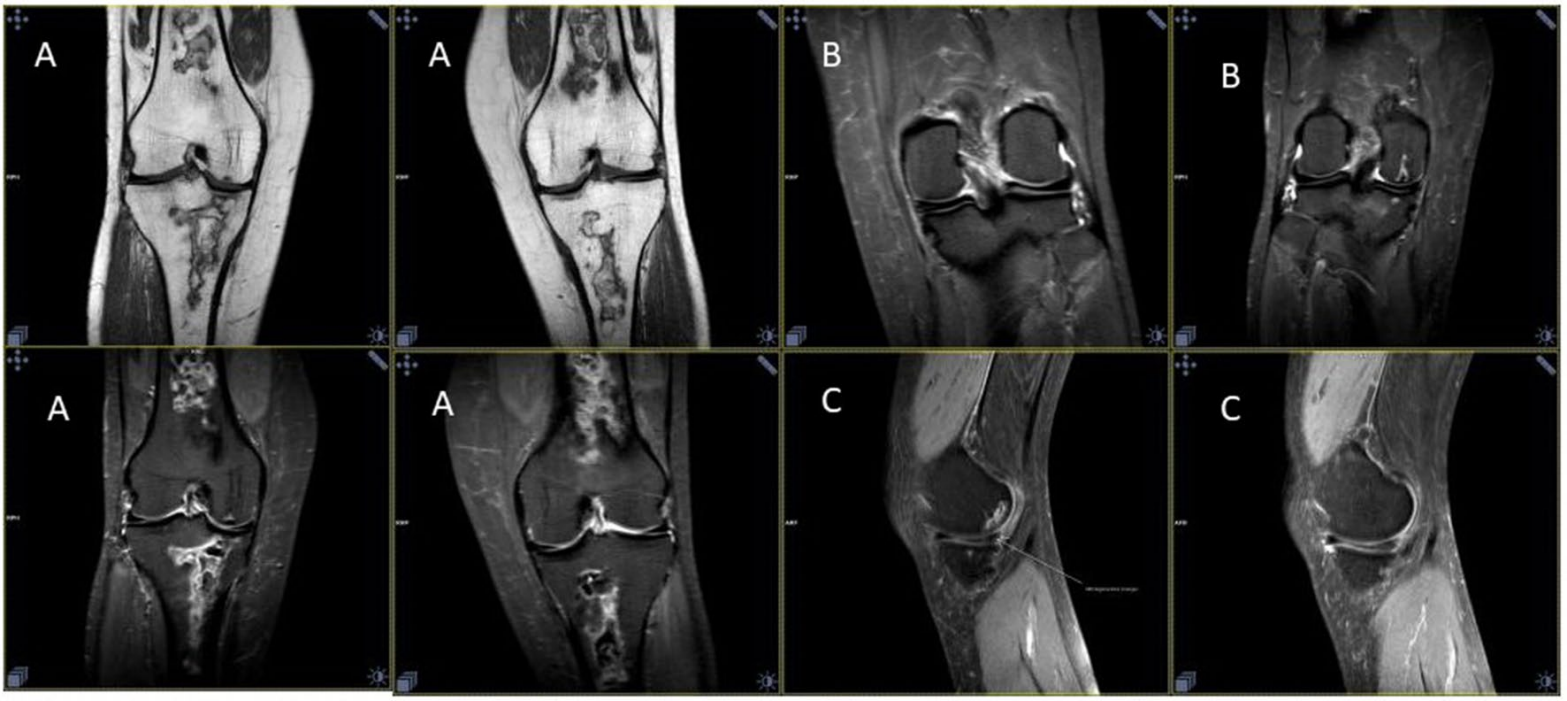
MRI of the knees. **A** Bilateral extensive regions of bone infarction in the shaft, epiphysis and metaphysis of femur and tibia. **B** Bilaterally 2nd grade chondropathy in the zones of femur condyle support. **C** Degenerative changes of medial meniscuses in the body and posterior horn as well as 2nd grade chondromalacia of the patella

A 45 year old female with non-treated lupus diagnosed 15 years earlier (no medical documentation) was admitted to the hospital with symptoms of active autoimmune disease. On admission she presented skin lesions of subacute lupus, pericardial effusion, autoimmune hemolytic anemia, leucopenia, low complement components’ levels, high concentration of dsDNA antibodies and active renal disease with daily proteinuria of 4,6 g/24 h, hematuria, creatinine 5,3 mg/dl, eGFR 9 ml/min, metabolic acidosis, hyperuricemia, hypocalcemia, hyperphosphatemia, deep vitamin D deficiency. Antiphospholipid antibodies were negative. CT scan of abdomen revealed nephrocalcinosis and abdominal lymphadenopathy. Due to active SLE with advanced renal insufficiency immunosuppressive treatment has been implemented (EUROLUPUS scheme), but renal function did not improve. Renal replacement therapy in the form of peritoneal dialysis was started. After one year the patient received a deceased donor renal transplant and underwent therapy with tacrolimus, mycophenolate mofetil and steroids. The graft was functioning well, and immunosuppressive medication concentrations were stable (Fig. 2). Approximately 2 years after transplantation she started complaining of increasing pain in both knees, most pronounced at night and after physical activity without morning stiffness. Additionally, she reported gradual weight loss (BMI 19) resulting from significantly decreased appetite, depressed mood, sleep disorders, vertigo, generalized weakness. Physical exam revealed tenderness and bilateral crepitus in knee joints without range of motion restriction, skin erythema, edema, or increased warmth of the knees. There were no pathological findings in the knees X-ray (Fig. 3). Dual energy X-ray absorptiometry (DXA) revealed osteoporosis. There were no other symptoms of active lupus. A diagnosis of osteoarthritis and osteoporosis in a patient with somatization symptoms due to depression was established, leading to new treatment strategy: antidepressant medication (duloxetine), anti-osteoporotic therapy (bisphosphonates), cartilage-metabolism enhancing medication with chondroitin sulphate associated with physiotherapy for a quadriceps strengthening program. Patient’s general condition improved, joint pain diminished. Subsequently the patient discontinued the therapy without consulting her physician. After one year, during the visit, the patient reported persistent, strong knee pain, hindering her everyday existence. Pain was described as constant, present throughout the day and night, still without morning predilection. As a self-relief method patient consumed maximal doses of nonsteroidal anti-inflammatory drugs, which consequently led to the rise of serum creatinine level. Again, physical examination revealed no abnormalities except bilateral crepitus in both knee joints. Assessed lupus activity level was low, the patient did not present depression symptoms. The immunosuppressive therapy at the time included tacrolimus 1 mg twice daily, 10 mg prednisone daily, and mycophenolic acid 720 mg twice daily (tacrolimus serum level 5,6 ng/mL) (Fig. 2). Laboratory results presented creatinine concentration 2,2 mg/dl (eGFR 24 ml/min), normocalcemia, normal phosphatemia, mildly increased alkaline phosphatase (115 U/l; N 41–108 U/l), parathormone 34.6 pg/ml (N 11–67 pg/ml). MRI of knee joints revealed bilateral extensive regions of bone infarction in the shaft, epiphysis and metaphysis of femur and tibia, chondropathy, degenerative changes of medial meniscuses in the body and posterior horn as well as chondromalacia of the patella (Fig. 4). MRI of the hips was normal. Avascular necrosis associated with osteoarthritis and chondropathy was recognized. Avocado soy unsaponifiables and analgesics (tramadol with paracetamol) were included in the treatment resulting in significant pain reduction and improvement of the patient's function. Immunosuppressive treatment was not modified, and low dose of prednisone was maintained to prevent graft rejection and exacerbation of lupus. Subsequent improvement in the clinical symptoms was archived.

## Discussion

Joint problems may appear at different times after the kidney transplantation (KT) and have diverse causes. End-stage renal insufficiency and chronic dialysis have been reported to contribute to a wide range of musculoskeletal and articular complications, but most of the symptoms improved after KT as a result of corticosteroid therapy. Our literature review revealed only two original publications focused directly on causes of joint pain in patients after kidney transplantation and one study based on MRI findings in kidney recipients with hip and knee pain (Table 1). None of them were restricted to patients with lupus. The recent paper by Haasan et al. provides an up-to-date review of the literature on causes of musculoskeletal disorders in renal transplant patients [2]. The authors concluded that bone loss ranged from 14 to 88%, calcineurin inhibitor pain syndrome (CIPS) ranged from 0.82% to 20.7%, while the prevalence of gout ranged from 7.6% to 22.37%. If we focus directly on knee joint pain, we find that among 82 patients in Kart-Köseoglu et al. group, 20% began to suffer joint pain after KT, 50% of them had knee pain, and 3.7% developed arthritis [1]. In Atallah et al. group, among 117 KT patients, 48 complained of knee pain [3]. Potential causes of knee pain, typical clinical symptoms, and management methods of each of the diagnoses have been presented in the Table 3.

**Table 3:** Differential diagnoses of chronic knee pain in lupus patient after renal transplantation

Disease	Etiology	Anamnesis	Clinical symptoms	Laboratory tests/imaging	Management
Lupus arthritis	Reoccurrence of active lupus after transplantation	Earlier diagnosis of lupus onset within approximately 4 years after transplantation	Join pain with morning stiffness; join effusion	Laboratory and immunological indicators of lupus	Modification of immunosuppressive treatment
Osteoarthritis	Degenerative changes, progressing with age, aggravated by weight and excessive physical activity (especially repetitive, high impact movements)	Worsening after physical activity stiffness after rest which resolves after a short warm-up activities. Previous trauma to the joint, history of elite sport may indicate early onset osteoarthritis. Obesity currently or in the anamnesis	Tenderness Crepitus during movement Sometimes joint effusion In advanced cases – deformations of joints, range of motion limitations Weakening of surrounding muscles (consequence of avoiding painful movements)	X-ray (narrowing of joint space, osteophytes) In case of suspected co-existing soft tissue abnormality MRI Normal lab tests	Physiotherapy Weight reduction Pain management
Chondromalacia	Imbalance of muscle strength, incorrect movement patterns, dispositioning of patella, previous trauma, female	Progressive character Previous trauma to the joint Sedentary lifestyle History of high impact sports (basketball, handball, volleyball, martial arts)	Usually valgus knees (however any deformation may cause cartilage changes) Tenderness of the patella upon movement and pressure Lateralized patella, patella alta or poorly mobile patella May lead to effusion	Usually physical examination is sufficient Ultrasound of the joint may be performed	Physiotherapy Arthroscopic management Good results of PRP therapy
Degenerative meniscus tear	Sequel of osteoarthritis	Progressive character of pain Usually medially Minor ‘trauma’ associated with the onset Feeling of locking in the knee Mainly present at bending movements – squatting, kneeling Worse after activity, diminishes with rest	Crepitation Positive meniscus tests Range of motion limitation Effusion	X-ray confirming osteoarthritis MRI should be considered	Arthroscopic management controversial (may facilitate further progression of osteoarthritis) Physiotherapy
Metabolic disturbance	Hormonal and electrolyte imbalance after renal transplantation	Onset clearly related to the surgical procedure No other significant issues such as for example trauma Non-specific character of pain	Findings of co-existing musculoskeletal abnormalities may be present but do not fully explain reported complaints	Parathormone, calcium, phosphate, vitamin D level Densitometry to confirm/exclude osteomalacia/osteoporosis	Proper supplementation of elements Early management of osteoporosis Moderate physical activity
Calcineurin inhibitor pain syndrome (CIPS)	Side effect of calcineurin inhibitor	Post-transplantation status (3 weeks-14 months after) Other diseases treated with calcineurin inhibitors (psoriatic arthritis, Still’s disease, Crohn’s disease, ulcerative colitis) Acute onset Symmetric character mainly lower extremities Aggravated by walking and standing resolves spontaneously with rest and elevation	No significant findings	Mildly elevated bone-specific alkaline phosphatase and calcium bone marrow edema in the MRI potentially increased serum drug levels	No specific management strategy avoid NSAIDS – might worsen the transplanted kidney function modification of immunosuppressive treatment (non-calcineurin agent) monitoring of CNI levels and adjusting the dose iloprost (prostacyclin analogue, therefore having vasodilation effect) might be effective
Avascular necrosis	Not fully understood; interruption of blood supply leading to cellular death	Onset usually 6–24 months after transplant Complaints in the major joints of lower and upper extremities (predominantly femoral head!) Pain of permanent character Worsened by weight-bearing Earlier diagnosis of lupus (risk factor) Corticosteroid treatment	No characteristic findings	MRI – bone marrow edema	Core decompression (sometimes with bone grafting) Surgical management – arthroplasty, core decompression, osteotomies, bone grafting Reduction or early withdrawal of corticosteroids

Joint involvement is one of the most common symptoms of active SLE. Based on the 2019 European League Against Rheumatism/American College of Rheumatology classification criteria for systemic lupus erythematosus, it is defined as either synovitis involving two or more joints, characterized by swelling or effusion, or tenderness in two or more joints and at least 30 min of morning stiffness [4]. Recurrence of active SLE has been reported both days and years after transplantation, with a median time to recurrence of approximately 4 years [5, 6]. The lack of clinical symptoms of arthritis and short time of morning stiffness suggested looking for another cause of the symptoms especially as there were no other signs of active SLE.

A spectrum of abnormalities related to chronic renal failure such as hyperphosphatemia, hypocalcemia, hyperparathyroidism, osteomalacia, osteopenia, and osteoporosis influence bone structure and trigger bone pain. During the first months after kidney transplant, there is a rapid bone loss mainly due to steroid treatment, but also due to deficiencies of vitamin D, continued secondary hyperparathyroidism, and impaired physical activity [7]. After the first year, patients may either continue to lose bone at a slower rate, stabilize, or even improve bone mineral density depending on numerous factors including medication usage, renal function, smoking, alcohol abuse, hypogonadism, aging, poor nutrition, and physical activity [7]. The presented patient had a low level of vitamin D, but her calcium-phosphate balance and PTH level were within normal ranges. Although the patient had symptoms of nephrocalcinosis in her own kidney, no recurrence was observed in the transplanted kidney. The cause of this condition was not explained in the patient.

Classical X-ray of our patients’ knee did not show any abnormalities, but MRI revealed the true causes of her complaints. Osteoarthritis (OA), chondromalacia and degenerative changes of meniscuses are one of the major causes of musculoskeletal pain [8, 9]. Tenderness and bilateral crepitus during movement, pain after exercises or prolonged sedentary position, ‘locking’ in the knee, sometimes accompanied by swelling and stiffness are main reported symptoms (see Table 3). In the Donmez et al. study, which analyzed MRI images in kidney transplant recipients with hip and knee pain with no history of trauma, avascular necrosis was found to be the most common etiology of hip pain (24 out of 57 patients) [10]. Intraarticular effusion was found to be the second reason for pain (18 patients), but tendinitis, bursitis and soft tissue abscess were also found. However, very interesting changes were found in the knee joints: among 24 patients, nine patients had degenerative joint disease, seven patients had chondromalacia, five had bone marrow edema, six had meniscal tears, six had ligament rupture and two had bone infarct [10]. The authors concluded that the most common etiology of hip pain in renal transplant recipients was avascular necrosis as expected but knee pain was explained by ligament pathology, meniscal tear, chondromalacia, or degenerative joint disease rather than osteonecrosis [10].

Avascular necrosis (AVN) of bone is a well-known complication of SLE even without renal involvement, that occurs in about 4–15% of patients [11, 12], even more if it is asymptomatic [13]. Of 1729 patients with systemic lupus erythematosus registered in the Toronto Lupus Clinic database, 234 (13.5%) developed symptomatic osteonecrosis, with hips and knees being most commonly affected [14]. Forty seven percent of the patients had multiple sites involved. In Turkish group of SLE patients reported by Sayarlioglu et al., 6% had avascular necrosis with femoral heads being the most common site of AVN (84% of patients), while the avascular knee changes were present in 27% of cases [15]. Shigemura et al. focused on incidence of osteonecrosis associated with corticosteroid therapy among different diseases [16]. They concluded that the incidence of osteonecrosis was significantly higher in SLE patients than in non-SLE patients (37% vs 21%). They also found that osteonecrosis of the femoral head occurred at a significantly higher rate in the non-SLE group than in the SLE group while knee joints were more often involved in SLE group. The reported in literature prevalence of knee avascular necrosis in patients with lupus is summarized in Table 2. This not fully understood pathology is defined as cellular death of bone components due to interruption of blood supply [17]. This complication used to develop in approximately one third of post-transplant patients, but the recent reports suggest that the use of steroid-sparing anti-calcineurin agents has reduced incidence rates to less than 5% [18–20]. In the study of Felten et al. the avascular necrosis was found in 4% among 805 kidney transplant recipients [19]. It usually appears 6–24 months after kidney transplant. Avascular necrosis commonly affects the femoral head, knee, shoulder, or elbow, however, among transplant recipients is commonly multifocal (extends to three or more separate anatomical sites). Pain associated with osteonecrosis is permanent, usually proximal, weight-dependent, and mainly localized in the hips.

Many theories regarding the pathophysiology of blood interruption have been proposed and include increased bone marrow pressure and intravascular occlusion of subchondral vessels by coagulation, fat emboli or thrombi [21].

Additionally corticosteroid-associated osteonecrosis has been observed in various diseases including systemic lupus, rheumatoid arthritis, asthma, inflammatory bowel diseases and organ transplantation. Steroid therapy, regardless of the original indication, presents a risk of development of multifocal osteonecrosis [22]. Of the 200 patients found to have multifocal osteonecrosis at study conducted by Flouzat-Lachaniette, all the patients had hip involvement at study entry and 60% had knee involvement. After an average time of 15 years of observation, only 17.5% patients developed new osteonecrosis lesions during the period study on a site where a contralateral lesion was present and mainly in the patients treated with peak doses (> 200 mg) of corticosteroids [22]. Calculated total dose of prednisone in our patients was about 24 g.

Several risk factors refer to the general population (corticosteroids, alcohol consumption, dyslipidemia, and hemostatic disorders), others are specific for the post-transplant population (persistent secondary hyperparathyroidism [19], preexisting bone disease, osteopenia and osteoporosis, a high body mass index (BMI), hipocalcemia [23]). Being overweight/obese, having pre-transplant diabetes or hyperparathyroidism at transplantation, developing acute rejection, and receiving higher cumulative corticosteroid doses were associated with AVN occurrence [19]. Meta-analysis performed by Hussein clearly indicated that steroids are strong risk factor associated with AVN in patients with SLE [24], while other risk factors, such as arthritis, neuropsychiatric manifestations of SLE, vasculitis, hypertension, serositis, and renal disease may be moderately associated with AVN. Sayarlioglu et al. in their group have noted that male gender and younger patients had more often AVN [15]. Meta-analysis performed by Nevskaya et al. indicated that high-dose corticosteroids and its’ side-effects (hypertension, Cushing’s, but not diabetes mellitus or hyperlipidemia) were associated with AVN, as was active SLE (cutaneous vasculitis, renal and neuropsychiatric manifestations, serositis, cytopenias) and Sjögren’s, Raynaud’s phenomenon, arthritis, cyclophosphamide (but not azathioprine mycophenolate mofetil, or methotrexate) and more damage (excluding musculoskeletal system) [13]. Avascular necrosis of the femur head and osteoporosis have been rarely reported in various studies as late complications in kidney transplant SLE recipients [25, 26], but some form of added risk could be expected.

Immunosuppression-related bone marrow edema syndrome also known as post-transplant bone marrow edema syndrome or calcineurin inhibitor pain syndrome (CIPS) was another potential cause of severe bone pain in reported patient. It was first mentioned in the early 1990s but finally recognized as a distinct post-transplant pain syndrome in 2001 by Grotz, et al. [27]. Since the first description, CIPS has been reported in patients after kidney, heart, lung, liver, pancreas, or bone marrow transplantation, but also in patients treated with calcineurin inhibitor due to psoriatic arthritis, Still’s disease, Crohn’s disease and ulcerative colitis. It is uncommon, painful side effect of calcineurin inhibitor use reported both after cyclosporine and tacrolimus treatment [28]. Patients usually present with sudden onset of severe, symmetric, bilateral pain in the lower extremities, usually involving the knees, feet, and the ankles. It usually appears in the first 3 weeks to 14 months after transplant. There are no specific abnormalities in physical examination, but the pain is worse with walking and standing and is reduced with rest and elevation of the legs. Despite sudden onset and severity, it usually resolves with rest within a few weeks to 10 months. Spontaneous resolution of the damage is not seen however, some initial changes may disappear. In laboratory tests mildly elevated bone-specific alkaline phosphatase and calcium before the onset of symptoms is reported in most of the patients [29]. They were also elevated in our patient. The pathophysiology is not clearly understood but it seems to be related to intra-osseous vasoconstriction and disturbed bone perfusion [30]. It leads to bone marrow edema, which is the most typical finding in MRI [10]. In some of the studies, it was postulated that pain in CIPS was correlated to elevated trough drug levels [27], which seems to be confirmed by observation that most patients experience symptomatic improvement with reduction of drug levels [31]. The most severe pain at night, the prolonged nature of the symptoms and the absence of bone marrow edema on MRI allowed the exclusion of CIPS in presented patient.

## Conclusion

In case of bone and joint pain establishing the right diagnosis is crucial for implementation of successful management. When dealing with an SLE patient after renal transplantation numerous potential causes need to be taken into consideration. However, it must always be considered that, as in the present case, there may be more than one cause of joint pain. Although avascular necrosis is a fairly common complication of chronic steroid therapy, it should be borne in mind that it is not only the hip joint that may be affected. Elements of anamnesis such as character of pain, aggravating and alleviating circumstances, time from the onset, together with laboratory findings and imaging examinations must be analyzed simultaneously to create a coherent picture of specific disorder.

## Data Availability

Not applicable.
